# Using a simple bedside assessment to predict hospital mortality in older adults with dementia: findings from the multicenter CHANGE Study

**DOI:** 10.1002/alz70858_103691

**Published:** 2025-12-25

**Authors:** Thiago J Avelino‐Silva, CHANGE Study Group

**Affiliations:** ^1^ University of California San Francisco, San Francisco, CA, USA, University of São Paulo Medical School, São Paulo, São Paulo, Brazil

## Abstract

**Background:**

Hospitalized older adults with dementia represent a population at high risk for adverse outcomes, including mortality. The Exton‐Smith Scale (ESS), originally designed to assess pressure sore risk, captures domains of physical condition, mental status, activity, mobility, and incontinence. However, its utility as a predictor of mortality in dementia has not been investigated. We aimed to determine whether ESS scores predict in‐hospital mortality among hospitalized older adults with dementia.

**Method:**

We conducted a prospective cohort study (the Creating a Hospital Assessment Network in Geriatrics [CHANGE] Study) across 43 hospitals in Brazil (*n* = 38), Angola (*n* = 1), Chile (*n* = 1), Colombia (*n* = 2), and Portugal (*n* = 1). We included hospitalized older adults (≥65 years) with dementia defined by a Clinical Dementia Rating (CDR≥1). Collected data included demographics, clinical history, ESS score (range: 5–20, with lower scores indicating higher risk), and hospital outcome (discharge vs. death). Multilevel mixed‐effects logistic regression, adjusting for age and sex, was used to evaluate associations between ESS and hospital mortality. We also explored interaction terms between ESS and CDR scores to assess effect modification by dementia severity.

**Result:**

Among the 766 participants (mean age 83±8 years; 65% female), 39% had mild dementia (CDR=1), 25% moderate dementia (CDR=2), and 36% severe dementia (CDR=3). The median ESS was 12 (interquartile range: 9–15), and overall in‐hospital mortality was 18%. We found that each 1‐point increase in ESS was associated with a 22% reduction in the odds of hospital mortality (OR=0.78, 95%CI=0.73–0.84; *p* <0.001). The model also demonstrated good discrimination (area under the ROC curve=0.81, 95%CI=0.77–0.84) and calibration. In the interaction model, the protective effect of higher ESS scores was strongest among those with mild dementia (CDR=1, OR=0.68, 95%CI=0.59–0.78; *p* <0.001).

**Conclusion:**

In a large cohort of hospitalized older adults with dementia, lower ESS scores were strongly associated with increased in‐hospital mortality. These findings suggest that a simple bedside scale initially designed to assess pressure sore risk may also be a valuable tool for identifying those at heightened risk of death. Incorporating ESS into routine dementia care could enhance clinical decision‐making and guide targeted interventions to improve hospital outcomes in this vulnerable population.

